# Ellagic Acid Enhances RSL3‐Induced Ferroptosis by Inhibiting the Nrf2/HO‐1 Signaling Pathway in Pancreatic Ductal Adenocarcinoma

**DOI:** 10.1096/fj.202602231R

**Published:** 2026-09-18

**Authors:** Jianhua Bai, Yihe Dai, Chern Ein Oon, Pingping Hu, Jia Luo, Jiang Han, Liman Yang, Amirabas Bostani, Zhenhao Fei, Yun Jin

**Affiliations:** ^1^ Department of Hepatopancreatobiliary Surgery. The First People's Hospital of Yunnan Province The Affiliated Hospital of Kunming University of Science and Technology Kunming Yunnan China; ^2^ Institute for Research in Molecular Medicine Universiti Sains Malaysia Minden Pulau Pinang Malaysia; ^3^ First Clinical Medical College Yunnan University of Chinese Medicine Kunming Yunnan China; ^4^ Department of Biology, Science & Research Institute Islamic Azad University Tehran Iran

**Keywords:** drug synergism, Kelch‐like ECH‐associated protein 1, MAP kinase signaling system, oxidative stress, pancreatic neoplasms

## Abstract

Pancreatic ductal adenocarcinoma (PDAC) exhibits profound therapeutic resistance due to redox adaptation, particularly through Nrf2/HO‐1 pathway activation. Ferroptosis induction via GPX4 inhibitors (e.g., RSL3) is promising but limited by adaptive antioxidant responses. Ellagic acid (EA), a natural polyphenol, may overcome this resistance, yet its role in modulating ferroptosis remains unexplored. *In vitro* studies used KRAS‐mutant and KRAS wild‐type PDAC cell lines (PANC‐1, BxPC‐3) treated with EA, RSL3, or both. Ferroptosis markers (iron, lipid ROS, MDA, GPX4), viability assays, and pathway analyses (Keap1/Nrf2/HO‐1, p38 MAPK) were evaluated. In vivo, antitumor efficacy was assessed in PANC‐1 xenografts. EA synergized with RSL3, reducing viability in PDAC cells (*p* < 0.001) and suppressing tumor growth in vivo (*p* < 0.001). Combination therapy amplified ferroptotic markers as increased intracellular iron, MDA, and lipid ROS, versus RSL3 alone, while GPX4 expression decreased. Ferroptosis specificity was confirmed via Fer‐1 rescue. Mechanistically, EA activated p38 MAPK, suppressing Nrf2 nuclear translocation and HO‐1 expression. Keap1 upregulation further enhanced Nrf2 degradation. In vivo, EA + RSL3 downregulated Nrf2/HO‐1 and elevated phospho‐p38 in tumors along with the induction of ferroptosis. EA potentiates RSL3‐induced ferroptosis in PDAC by disrupting the p38/Nrf2/HO‐1 axis and elevating Keap1. This natural compound‐based strategy overcomes redox‐driven resistance, offering a translatable approach for PDAC models.

## Graphical abstract

Ellagic acid potentiates RSL3‐induced ferroptosis in PANC‐1 by suppressing the Nrf2/HO‐1 antioxidant defense axis via p38 MAPK activation and Keap1 upregulation. In PDAC cells, constitutive Nrf2/HO‐1 signaling establishes a robust antioxidant shield (GPX4, HO‐1) that limits lipid ROS accumulation and confers ferroptosis resistance. Co‐treatment with ellagic acid (EA) and RSL3 activates p38 MAPK phosphorylation, which suppresses Nrf2 nuclear translocation and HO‐1 expression, while concomitantly upregulating Keap1 to promote Nrf2 degradation. This dual inhibition dismantles the antioxidant defense barrier, leading to GPX4 downregulation, accumulation of intracellular iron (Fe^2+^) and malondialdehyde (MDA), and elevated lipid peroxidation, culminating in ferroptotic cell death. In vivo, EA + RSL3 combination therapy significantly suppresses PANC‐1 xenograft tumor growth compared to monotherapy or control groups, demonstrating the translational potential of this natural compound‐based strategy to overcome redox‐driven therapeutic resistance in PDAC.

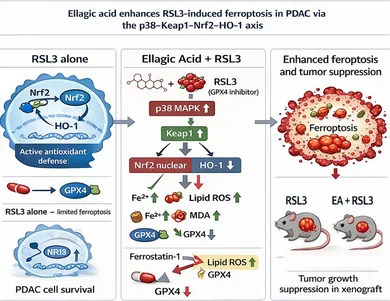

## 1 Introduction

Pancreatic ductal adenocarcinoma (PDAC), the most predominant type of pancreatic cancer, remains one of the most lethal malignancies worldwide [1]. The disproportionate lethality of PDAC is primarily attributed to its aggressive biological behavior, frequent late‐stage diagnosis, and poor responsiveness to conventional therapeutic modalities [2]. Despite advancements in systemic chemotherapy, including gemcitabine‐based regimens and the multi‐agent FOLFIRINOX (fluorouracil, leucovorin, irinotecan, and oxaliplatin) protocol, the median survival for individuals with advanced PDAC seldom exceeds 12 months, and the 5‐year survival rate remains below 10% [3, 4, 5]. Therapeutic resistance in PDAC is driven by both tumor‐intrinsic and microenvironmental factors. Hallmarks of PDAC include a dense, fibrotic stroma, hypoxic tumor microenvironments, and robust activation of redox‐regulating pathways that collectively confer resistance to oxidative stress and apoptosis [6, 7, 8, 9, 10]. Notably, persistent activation of the nuclear factor erythroid 2‐related factor 2 (Nrf2) pathway plays a critical role in maintaining cellular antioxidant defense mechanisms, thereby facilitating survival under therapeutic pressure [11, 12]. Although newer drug combinations, such as nab‐paclitaxel with gemcitabine, have shown incremental benefits, these gains are often transient due to the rapid emergence of chemoresistance [13, 14, 15, 16, 17].

One promising avenue involves targeting non‐apoptotic forms of regulated cell death to overcome resistance mechanisms [18, 19]. Ferroptosis, a recently characterized, iron‐dependent form of cell death distinguished by lipid peroxidation and depletion of antioxidant systems, has emerged as a compelling candidate [20]. Unlike apoptosis or necroptosis, ferroptosis is initiated by the accumulation of lipid hydroperoxides and iron‐catalyzed Fenton reactions, which are normally mitigated by the glutathione, glutathione peroxidase 4 (GPX4) axis, and CoQ_10_‐dependent mechanisms [19, 20, 21]. Pharmacologic inducers of ferroptosis, such as the GPX4 inhibitor RSL3, have demonstrated preclinical efficacy in inducing lipid reactive oxygen species (ROS) and ferroptotic cell death in resistant tumor models, including PDAC [22]. However, their therapeutic utility is frequently constrained by the activation of adaptive resistance pathways, notably Nrf2 signaling [23, 24]. Nrf2 is a master regulator of oxidative stress responses, modulating the transcription of detoxification and antioxidant genes such as heme oxygenase‐1 (HO‐1). While HO‐1 can influence ferroptosis in a context‐dependent manner, its upregulation generally reinforces resistance by enhancing antioxidant capacity and facilitating iron sequestration [25, 26, 27, 28].

In parallel, there is growing interest in natural compounds with multi‐targeted anticancer activities. Ellagic acid (EA), a polyphenolic antioxidant found in pomegranates, berries, and nuts, has demonstrated a spectrum of antitumor effects, including the induction of apoptosis, inhibition of angiogenesis and inflammation, suppression of proliferation, and blockade of metastatic progression [29, 30, 31]. In PDAC models, EA has been shown to reduce tumor burden in PANC‐1 xenografts, induce G₁ cell cycle arrest, and suppress survival pathways such as NF‐κB and COX‐2 [32, 33, 34, 35]. Emerging data suggest that EA modulates redox‐sensitive signaling cascades, particularly those involving Nrf2 and mitogen‐activated protein kinases (MAPKs) [36, 37].

Despite these observations, the role of EA in modulating ferroptosis, particularly in combination with GPX4 inhibitors such as RSL3 in PDAC, remains underexplored. Further investigation is warranted to elucidate the mechanistic interplay among EA, p38 MAPK activation, Nrf2 suppression, and ferroptosis sensitization in PDAC. Accordingly, this study aimed to investigate the impact of EA on RSL3‐induced ferroptosis in PDAC cell lines, specifically PANC‐1 and BXPC‐3. Furthermore, the research evaluated the therapeutic potential of this combination approach in a xenograft mouse model. By elucidating the molecular mechanisms involved, the study seeks to identify a promising strategy to enhance ferroptosis and potentially overcome therapeutic resistance in PDAC.

## 2 Materials and Methods

### 2.1 Cell Lines and Culture Conditions

Human PDAC cell lines PANC‐1 and BxPC‐3, along with the immortalized normal human pancreatic ductal epithelial cell line HPDE6‐C7 (H6C7), were sourced from the American Type Culture Collection (ATCC, USA). Upon receipt, the cells were expanded and verified through short tandem repeat (STR) profiling to confirm their identity. Routine screening for mycoplasma contamination was conducted using the MycoAlert Mycoplasma Detection Kit (Lonza). Cell lines were propagated in high‐glucose Dulbecco's Modified Eagle Medium (DMEM; Gibco), enriched with 10% (v/v) heat‐inactivated fetal bovine serum (FBS; Gibco), 100 U/mL penicillin, and 100 μg/mL streptomycin (Gibco). Cell incubation was carried out at 37°C in a humidified environment of 5% CO_2_. A subculturing protocol was followed every 2–3 days utilizing 0.25% trypsin–EDTA (Gibco) to dissociate the cells, ensuring confluency remained below 80%.

### 2.2 Reagent Preparation and Treatment Design

Ellagic acid (EA; ≥ 98% purity) and RSL3 were obtained from Sigma‐Aldrich. Stock solutions were prepared by dissolving EA (10 mM) and RSL3 (1 mM) in dimethyl sulfoxide (DMSO; Sigma‐Aldrich). These solutions were sterile‐filtered using 0.22 μm membranes and stored at −20°C, protected from light. Prior to each experiment, stock solutions were diluted to their target working concentrations (EA: 0, 10, 20, 50, 100 μM; RSL3: 0, 0.25, 0.5, 1, 2 μM; or their combination) using fresh culture medium. The final concentration of solvents in all treatments was maintained below 0.1% (v/v). The control group was treated with DMSO as a vehicle control. For all assays, cells were seeded and allowed to adhere overnight before initiating treatment. In cell viability assays, 5 × 10^3^ cells per well were plated in 96‐well plates, with three technical replicates per condition. For apoptosis and lipid peroxidation analyses, 1 × 10^5^ cells per well were seeded in six‐well plates, using three independent biological replicates. For gene expression studies, 1 × 10^6^ cells were plated in 10 cm culture dishes, also in triplicate. All experimental treatments were conducted for 24 h at 37°C unless stated otherwise.

### 2.3 Cell Viability Assay

After 24 h of treatment, cellular metabolic activity was evaluated using both the MTT assay and Cell Counting Kit‐8 (CCK‐8; Dojindo) methods. For the MTT assay, 10 μL of MTT solution was added to each well, followed by a 2‐h incubation at 37°C. Subsequently, 100 μL of solubilization buffer was added to dissolve the resulting formazan crystals. Absorbance was then measured at 570 nm using a microplate spectrophotometer (BioTek ELx800). In the CCK‐8 assay, 10 μL of the reagent was dispensed into each well of a 96‐well plate, and the plate was incubated at 37°C for 2 h. Absorbance was recorded at 450 nm using a microplate reader, with wells containing only medium used as blanks. Cell viability was determined by normalizing the absorbance values of treated wells to the mean absorbance of the vehicle control.

### 2.4 Quantification of Malondialdehyde (MDA) and Lipid ROS Levels

Subsequent to the treatment, cells were rinsed with PBS, trypsinized, and centrifuged (10,000×*g*, 10 min). The pellet was lysed (50 mM phosphate buffer, pH 7.0), sonicated (2 min, cold), and centrifuged again to collect the supernatant. MDA, a key biomarker of lipid peroxidation, was measured via a colorimetric assay. Briefly, 100 μL of cellular extract was mixed with 400 μL of thiobarbituric acid (TBA) reagent (0.375% TBA, 15% trichloroacetic acid, and 0.25 M HCl) and incubated at 95°C for 30 min. After rapid cooling, the mixture was centrifuged at 8,000×*g* for 15 min (4°C), and the absorbance of the pink supernatant was measured at 532 nm. MDA concentrations were determined using a tetraethoxypropane (TEP) standard curve and expressed as μmol/mg protein.

For lipid ROS measurement, treated cells were washed twice with ice‐cold phosphate‐buffered saline (PBS), harvested by scraping, and centrifuged at 1,000×*g* for 10 min at 4°C. The cell pellet was resuspended in 200 μL of assay‐specific lysis buffer (50 mM Tris–HCl, pH 7.4, containing 150 mM NaCl and 1% Triton X‐100) and homogenized via sonication (3 pulses of 10 s on ice). The homogenate was centrifuged at 12,000×*g* for 15 min at 4°C to remove insoluble debris. Lipid ROS levels were quantified using the Lipid Peroxidation Colorimetric Assay Kit (Cayman Chemical, catalog No. 705003), per the manufacturer's instructions. A standard curve was generated using 1,1,3,3‐tetramethoxypropane (0–20 μM). The concentrations of lipid ROS were standardized against total protein content, which was measured using a Bradford assay (Bio‐Rad). The results are presented as nmol/mg protein. All samples were analyzed in triplicate.

### 2.5 Intracellular Iron Levels

Total iron levels in cell culture samples were quantified using the Invitrogen Cell Total Iron Colorimetric Assay Kit (Catalog #EEA009), in accordance with the manufacturer's protocol. Following two washes in ice‐cold PBS, the cells were centrifuged, harvested, and lysed using a 0.1% Triton X‐100 solution in PBS. The lysate was then homogenized via sonication and centrifuged (12,000×*g*, 10 min, 4°C) to remove insoluble debris. The resulting supernatant was diluted as needed to ensure iron concentrations remained within the assay's linear detection range (0.4–50 μmol/L). Total iron content was subsequently determined in accordance with the manufacturer's instructions.

### 2.6 Quantitative Reverse Transcription PCR (RT‐qPCR)

Total RNA was isolated from treated cells using TRIzol Reagent (Invitrogen) according to the manufacturer's protocol. RNA purity and concentration were assessed using a NanoDrop 2000 spectrophotometer (Thermo Fisher Scientific) and gel electrophoresis. cDNA was synthesized from 1 μg of total RNA via reverse transcription, employing the PrimeScript RT Reagent Kit (Takara Bio). Quantitative PCR (qPCR) was performed on a CFX96 Real‐Time PCR Detection System (Bio‐Rad) with SYBR Premix Ex Taq II (Takara Bio). Each 20 μL reaction mixture contained 1× SYBR Green master mix, 200 nM of forward and reverse primers, and 2 μL of cDNA (diluted 1:10). The thermal cycling protocol involved an initial 1‐min denaturation at 95°C, then 40 cycles of 15 s at 95°C, 15 s at 60°C, and 20 s at 72°C. Primer specificity was confirmed by melt curve analysis. The 2^−ΔΔCt^ method was employed to calculate relative gene expression, utilizing β‐actin as the endogenous reference gene (Table 1). All qPCR reactions were conducted in technical triplicate to ensure reproducibility.

**TABLE 1:** Primer sequences.

Primer	Sequence
β Actin forward	5′‐CACCATTGGCAATGAGCGGTTC‐3′
β‐Actin reverse	5′‐AGGTCTTTGCGGATGTCCACGT‐3′
GPX4 forward	5′‐ACAAGAACGGCTGCGTGGTGAA‐3′
GPX4 reverse	5′‐GCCACACACTTGTGGAGCTAGA‐3′
Nrf2 forward	5′‐CACATCCAGTCAGAAACCAGTGG‐3′
Nrf2 reverse	5′‐GGAATGTCTGCGCCAAAAGCTG‐3′
KEAP1 forward	5′‐CAACTTCGCTGAGCAGATTGGC‐3′
KEAP1 reverse	5′‐TGATGAGGGTCACCAGTTGGCA‐3′
p38 MAPK forward	5′‐GAAAGAGTGGCAACCTGCCTTC‐3′
p38 MAPK reverse	5′‐GCACCAAGTTTTACTACATCTGCC‐3′
HO‐1 forward	5′‐CCAGGCAGAGAATGCTGAGTTC‐3′
HO‐1 reverse	5′‐AAGACTGGGCTCTCCTTGTTGC‐3′
si‐Nrf2‐1 forward	5′‐UCUUCAUCACGUAGCAUGCTT‐3′
si‐Nrf2‐1 reverse	5′‐GCAUGCUACGUGAUGAAGATT‐3′
si‐Nrf2‐2 forward	5′‐GGAAGAAGAAUGACCUCAATT‐3′
si‐Nrf2‐2 reverse	5′‐UUGAGGUCAUUCUUCUUCCTT‐3′

### 2.7 siRNA‐Mediated Gene Silencing

To examine the functional role of Nrf2, a transfection was performed using 50 nM of either Nrf2‐targeting siRNA or a non‐targeting control siRNA (GenePharma) with Lipofectamine RNAiMAX (Invitrogen), following the manufacturer's instructions. In brief, siRNA–lipid complexes were prepared in Opti‐MEM medium (Gibco) and incubated at room temperature for 20 min before being added dropwise to cells at approximately 50% confluence and cultured in antibiotic‐free DMEM. After 6 h of transfection, the medium was replaced with a complete growth medium, and cells were further incubated for 48 h to ensure maximal gene silencing. Knockdown efficiency was validated via RT‐qPCR and ELISA analysis before proceeding with subsequent treatments.

### 2.8 Enzyme‐Linked Immunosorbent Assay

Protein levels of GPX4 (Abcam, #ab304936), Nrf2 (Abcam, #ab277397), HO‐1 (Abcam, #ab207621), Keap1 (FineTest, #EH4240), and phospho‐Thr180 p38 MAPK (Boster, #EKC2484) were quantified using commercial ELISA kits according to the manufacturers' protocols.

### 2.9 Animal Care and Xenograft Establishment

Male athymic BALB/c nude mice (RRID:IMSR_RJ: BALB‐C‐NUDE, 6–8 weeks old, 18–20 g) were maintained under specific pathogen‐free (SPF) conditions. Housing conditions consisted of a 12‐h light/dark cycle, a temperature of 22°C ± 2°C, and ad libitum provision of standard rodent diet and water. Prior to experimentation, the mice were acclimated for 1 week to ensure physiological stability. To establish xenograft tumors, PANC‐1 cells in the logarithmic growth phase were collected, rinsed twice with ice‐cold PBS, and resuspended at a density of 1 × 10^7^ cells/mL in a 1:1 solution of PBS and growth factor‐reduced Matrigel. Subsequently, a 100 μL aliquot of the cell suspension (containing 1 × 10^6^ cells) was administered via subcutaneous injection into the right flank of each mouse using a 27‐gauge needle.

Sample size (*n* = 6 per group) was determined a priori based on a power analysis (80% power, *α* = 0.05) using data from a preliminary experiment (mean tumor volume difference of 150 mm^3^, standard deviation of 45 mm^3^). Mice were included in the study if the xenograft tumor volume reached 80–120 mm^3^ with regular shape and no ulceration. Exclusion criteria were defined prior to randomization as follows: tumor volume < 80 mm^3^ or > 120 mm^3^, absence of visible tumor take, signs of necrosis or ulceration over the tumor site, or any systemic illness (e.g., lethargy, hunched posture, or > 20% body weight loss before treatment initiation). No animals met these exclusion criteria during the experiment. Animals were monitored twice daily for signs of distress, including piloerection, lethargy, hunched posture, reduced mobility, or labored breathing. Humane endpoints were predefined as follows: (1) tumor volume exceeding 1500 mm^3^, (2) tumor ulceration or bleeding, (3) body weight loss > 20% of baseline for two consecutive days, (4) inability to reach food or water, or (5) persistent signs of severe pain or distress not alleviated by analgesia. Any animal reaching a humane endpoint would be euthanized immediately via CO_2_ inhalation; however, no animal met these endpoints during the study.

### 2.10 Treatment Regimen and Monitoring

Once the tumor volumes reached approximately 100 mm^3^ (when xenograft tumors reached a volume between 80 and 120 mm^3^), mice were considered eligible for enrollment, measured using digital calipers and calculated as length × width^2^ × 0.5, mice were randomly allocated into four experimental groups (*n* = 6 per group) using a computer‐generated random number sequence (Random Allocation Software, version 2.0). The allocation sequence was concealed in sealed opaque envelopes, which were opened immediately before the first treatment administration. The treatment groups were: (1) Vehicle control group (Con, received daily intraperitoneal (i.p.) injections of 5% DMSO in saline); (2) EA monotherapy group (EA, administered 50 mg/kg EA in 5% DMSO/saline via daily i.p. injections); (3) RSL3 monotherapy group (RSL3, treated with 10 mg/kg RSL3 in 5% DMSO/saline via i.p. injections every other day); (4) Combination therapy group (EA + RSL3, received both EA (50 mg/kg daily) and RSL3 (10 mg/kg every other day) using the same vehicle formulations and administration routes). Animal treatment was performed by an investigator blinded to group allocation. Tumor measurements, daily monitoring, tissue processing, and all subsequent outcome assessments (ELISA, RT‐qPCR, Western blot) were conducted by investigators blinded to group identities until the final data analysis was completed. Blinding was maintained by coding all samples with numeric identifiers unrelated to treatment groups. Tumor dimensions and body weights were recorded at the end of treatments. Additionally, the animals were monitored daily for signs of distress, behavioral changes, or excessive weight loss (> 20% of baseline body weight).

### 2.11 Post‐Treatment Tissue Processing and Analysis

One day following the final treatment, mice were humanely euthanized via CO_2_ inhalation. Excised tumors were briefly rinsed in ice‐cold PBS, blotted dry, and divided into two portions for the downstream analyses described.

First, approximately 30 mg of tumor tissue was immediately placed in 1 mL of TRIzol reagent. The tissue was minced on ice, homogenized using a Dounce homogenizer, and processed for RNA extraction, followed by RT‐qPCR as described in the in vitro protocol. Second, approximately 50 mg of tumor tissue was snap‐frozen in liquid nitrogen and stored at −80°C for subsequent protein analysis.

### 2.12 Western Blot Analysis

For western blot analysis, protein samples (20–30 μg per lane) were mixed with 4× Laemmli buffer containing β‐mercaptoethanol, denatured by boiling for 5 min, and resolved on 10% SDS–PAGE gels. Following separation, the proteins were transferred to PVDF membranes (Millipore) at 100 V for 90 min in a transfer buffer. Membranes were blocked for 1 h at room temperature in 5% non‐fat dry milk prepared in Tris‐buffered saline containing 0.1% Tween‐20 (TBST) for 1 h at room temperature. Primary antibody incubation was performed overnight at 4°C using dilutions of 1:1000 for Nrf2 (Affinity Biosciences Cat# AF0639, RRID:AB_2833793), HO‐1 (Affinity Biosciences Cat# DF6391, RRID:AB_2838354), Keap1 (Affinity Biosciences Cat# AF5266, RRID:AB_2837752), p38 MAPK (Affinity Biosciences Cat# AF6455, RRID:AB_2846173), phospho‐p38 MAPK (Affinity Biosciences Cat# AF3457, RRID:AB_2834895), and β‐actin (Sigma‐Aldrich Cat# A5441, RRID:AB_476744). After three washes in TBST, the membranes were incubated for 1 h at room temperature with HRP‐conjugated secondary antibodies diluted 1:5000. Enhanced chemiluminescence (ECL; Bio‐Rad) was used to visualize immunoreactive bands on a ChemiDoc MP System (Bio‐Rad). Quantitative analysis of band intensity was conducted using ImageJ software, with target protein levels normalized to β‐actin as a loading control.

### 2.13 Statistical Analysis

All quantitative data are presented as mean ± standard deviation (SD). For in vitro experiments, comparisons among multiple treatment groups were assessed using the one‐way analysis of variance (ANOVA) with Tukey's post hoc test. Tumor growth curves from in vivo studies were evaluated via two‐way ANOVA, incorporating Bonferroni correction for repeated measurements. RT‐qPCR and Western blot densitometry data derived from in vivo samples were analyzed using one‐way ANOVA followed by Tukey's post hoc test. All statistical analyses were conducted in GraphPad Prism version 8.0 (RRID:SCR_002798). Quantitative analysis of band intensity was conducted using ImageJ software (RRID:SCR_003070), and a *p* < 0.05 deemed statistically significant.

## 3 Results

### 3.1 Combination of EA and RSL3 Reduces Viability in PDAC Cell Lines

**FIGURE 1:**
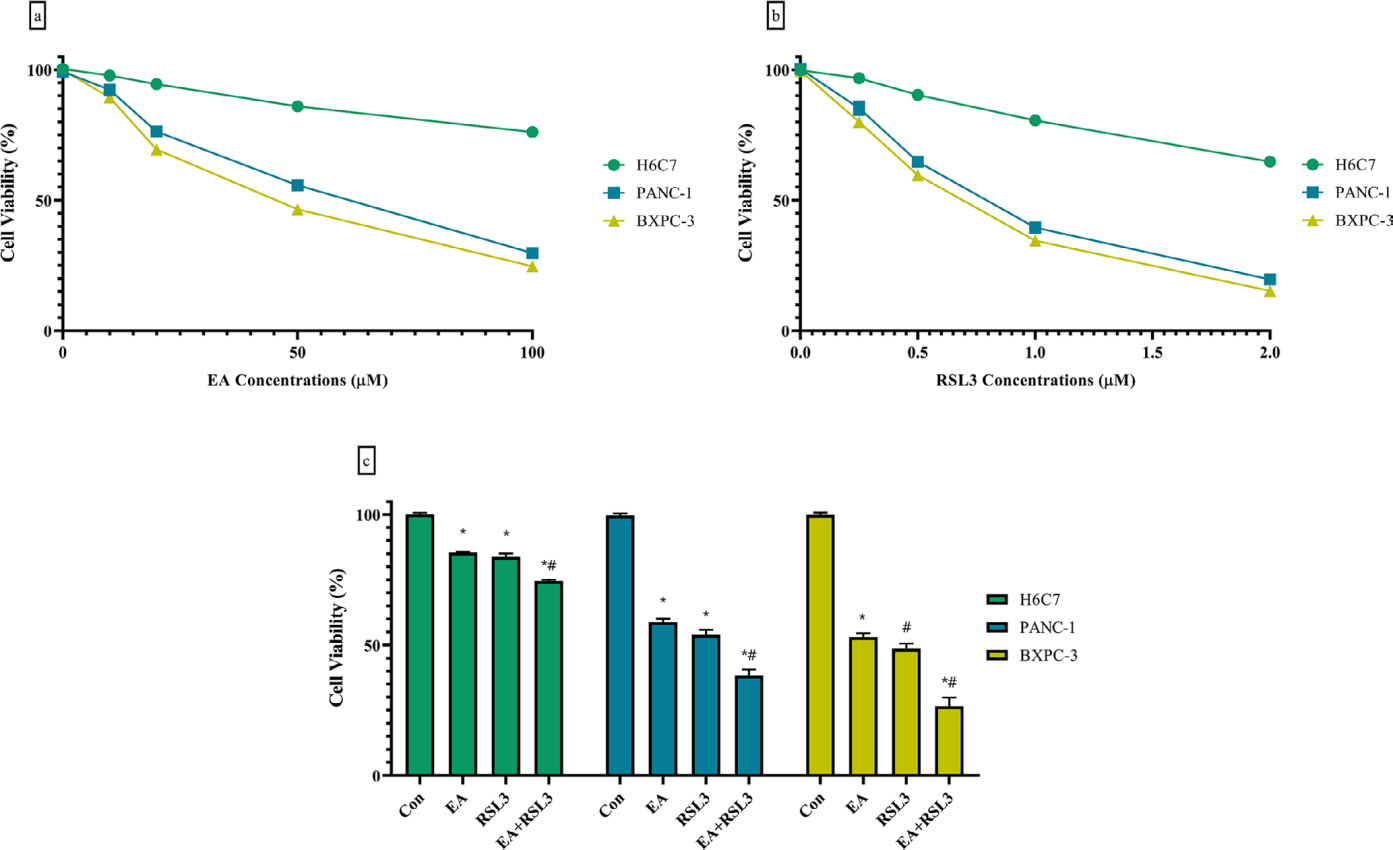
The effects of RSL3 on cell viability in PDAC cell lines were enhanced by EA. (a) MTT assay results following treatment with various concentrations of EA; (b) MTT assay results following treatment with different concentrations of RSL3; (c) Assessment of cell viability after treatment with EA (50 μM) and RSL3 (1 μM) either individually or in combination. Data are presented as mean ± SD. A *p* < 0.05 was deemed significant; *: Indicates a significant difference from controls; *#: Indicates a significant difference when compared to both control and RSL3 groups.

To evaluate the cytotoxic effects of EA and RSL3 alone or in combination, two PDAC cell lines (PANC‐1, BxPC‐3) and one normal pancreatic ductal epithelial cell line (H6C7) were treated with escalating concentrations of EA (0–100 μM) or RSL3 (0–2 μM) for 24 h. MTT assay revealed that monotherapy with EA (Figure 1a) or RSL3 (Figure 1b) demonstrated dose‐dependent cytotoxicity across all cell lines. For subsequent combination experiments, EA (50 μM) and RSL3 (1 μM) were selected based on their moderate individual efficacy (EA at 50 μM reduced viability to ~45%–58% in PANC‐1 and BxPC‐3, while RSL3 at 1 μM reduced viability to ~32%–41% in these cell lines), ensuring submaximal doses to avoid overt toxicity and allow observation of synergistic effects. The enhanced efficacy of the combination in PANC cells aligns with prior reports linking RAS mutations to ferroptosis sensitivity, suggesting that co‐targeting antioxidant pathways (via EA) and GPX4 inhibitors (via RSL3) may exploit metabolic vulnerabilities in these cells [38, 39]. Combination therapy with EA (50 μM) and RSL3 (1 μM) significantly reduced cell viability, measured by the CCK‐8 assay, compared to monotherapy across all tested cell lines (Figure 1c). These data suggest that co‐treatment with EA and RSL3 induced pronounced synergistic cytotoxicity, particularly in PANC‐1 cells.

### 3.2 EA Enhances RSL3‐Induced Ferroptosis in PDAC Cells

**FIGURE 2:**
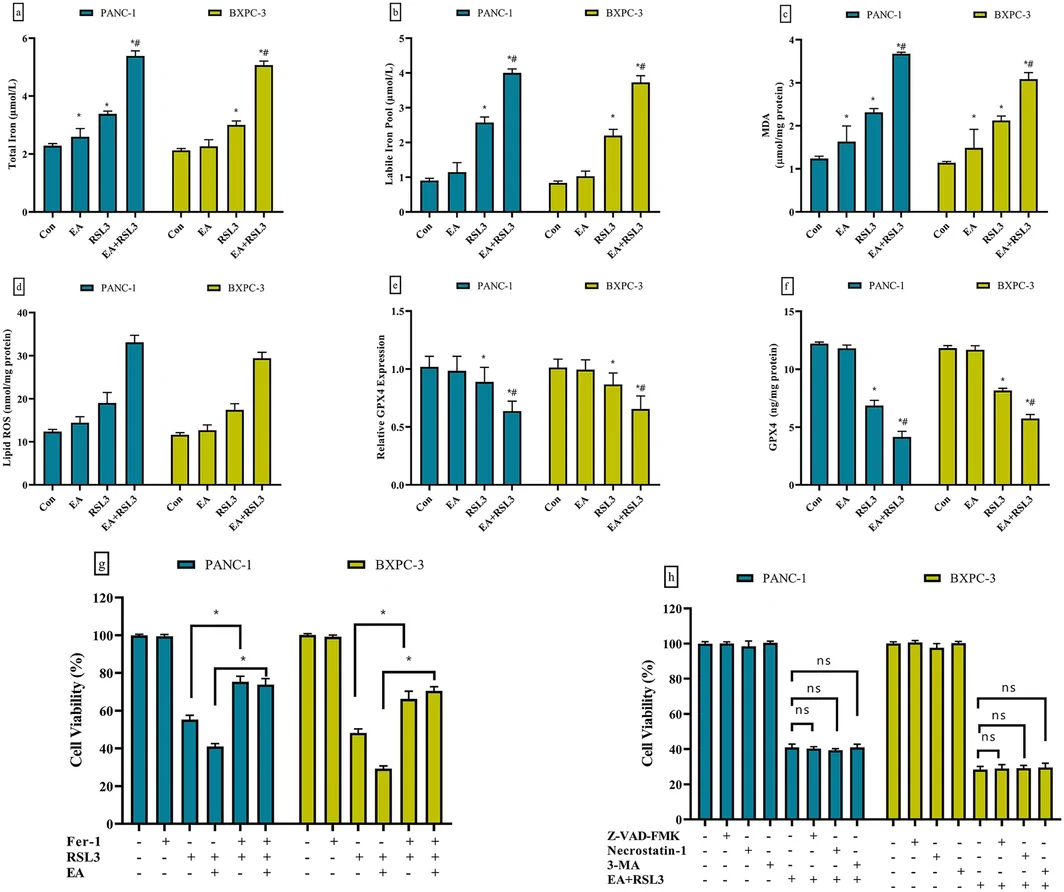
EA promotes RSL3‐induced ferroptosis in  PDAC cells. (a) Measurement of total iron content; (b) Evaluation of the chelatable iron pool; (c) Analysis of MDA levels; (d) Assessment of lipid ROS levels; (e) GPX4 mRNA expression levels; (f) GPX4 protein expression levels; (g) Viability rescue experiments with Fer‐1, PANC‐1, and BxPC‐3 cells treated with EA (50 μM) or RSL3 (1 μM) in the presence or absence of Fer‐1 (1 μM), with cell viability assessed using the CCK‐8 assay; (h) No viability rescue observed with apoptosis, necroptosis, or autophagy inhibitors, where cells were treated with EA (50 μM) and RSL3 (1 μM) in conjunction with Z‐VAD‐FMK (10 μM), necrostatin‐1 (10 μM), or 3‐MA (60 μM), and cell viability was evaluated via the CCK‐8 assay. Data are presented as mean ± SD. A *p* < 0.05 was considered significant; *: Indicates a significant difference from controls or the specified group; *#: Indicates a significant difference compared to both control and RSL3 groups; ns, not significant.

To investigate whether ferroptosis contributed to the cytotoxic effects of EA and RSL3, key ferroptosis markers, including intracellular iron levels, lipid peroxidation, lipid ROS, and GPX4 gene expression and protein levels, were assessed in KRAS‐mutant and KRAS wild‐type (PANC‐1 and BxPC‐3) cells. Intracellular total iron levels increased synergistically in combination‐treated cells (PANC‐1: 5.45 ± 0.21 μmol/L; BxPC‐3: 4.95 ± 0.13 μmol/L) versus RSL3 alone (*p* < 0.01, Figure 2a). Chelatable iron, indicative of the labile iron pool, also rose markedly in co‐treated cells compared to RSL3 monotherapy (*p* < 0.01, Figure 2b). Co‐treatment with EA and RSL3 significantly elevated MDA levels in both PANC‐1 (3.71 ± 0.03 μmol/mg protein) and BxPC‐3 (3.36 ± 0.15 μmol/mg protein) compared to monotherapy (*p* < 0.01, Figure 2c). Similarly, lipid ROS levels, a direct indicator of membrane lipid peroxidation, were quantified, and EA + RSL3 co‐treatment triggered a pronounced accumulation of lipid ROS in both PDAC cell lines, significantly surpassing monotherapy effects (Figure 2d, *p* < 0.001). GPX4, a critical ferroptosis defense enzyme, was downregulated at both mRNA and protein levels. Combination therapy reduced *GPX4* expression in both PDAC cell lines compared to RSL3 alone (*p* < 0.001, Figure 2e). Similarly, GPX4 protein levels decreased to 4.71 ± 0.35 ng/mg (PANC‐1) and 5.96 ± 0.42 ng/mg (BxPC‐3) with EA + RSL3 versus RSL3 monotherapy (*p* < 0.01, Figure 2f).

To confirm ferroptosis as the primary death mechanism, cells were co‐treated with inhibitors of ferroptosis (Fer‐1), apoptosis (Z‐VAD‐FMK), necroptosis (necrostatin‐1), or autophagy (3‐MA). As shown in Figure 2g, Fer‐1 significantly rescued viability in combination‐treated PANC‐1 (71.43% ± 2.15% vs. 41.37% ± 1.89%; *p* < 0.001) and BxPC‐3 (72.55% ± 2.34% vs. 29.28% ± 1.76%; *p* < 0.001). In contrast, Z‐VAD‐FMK, necrostatin‐1, and 3‐MA failed to reverse cytotoxicity (*p* > 0.05, Figure 2h).

### 3.3 EA and RSL3 Synergistically Modulated Keap1/Nrf2/HO‐1 Axis in PDAC Cells

**FIGURE 3:**
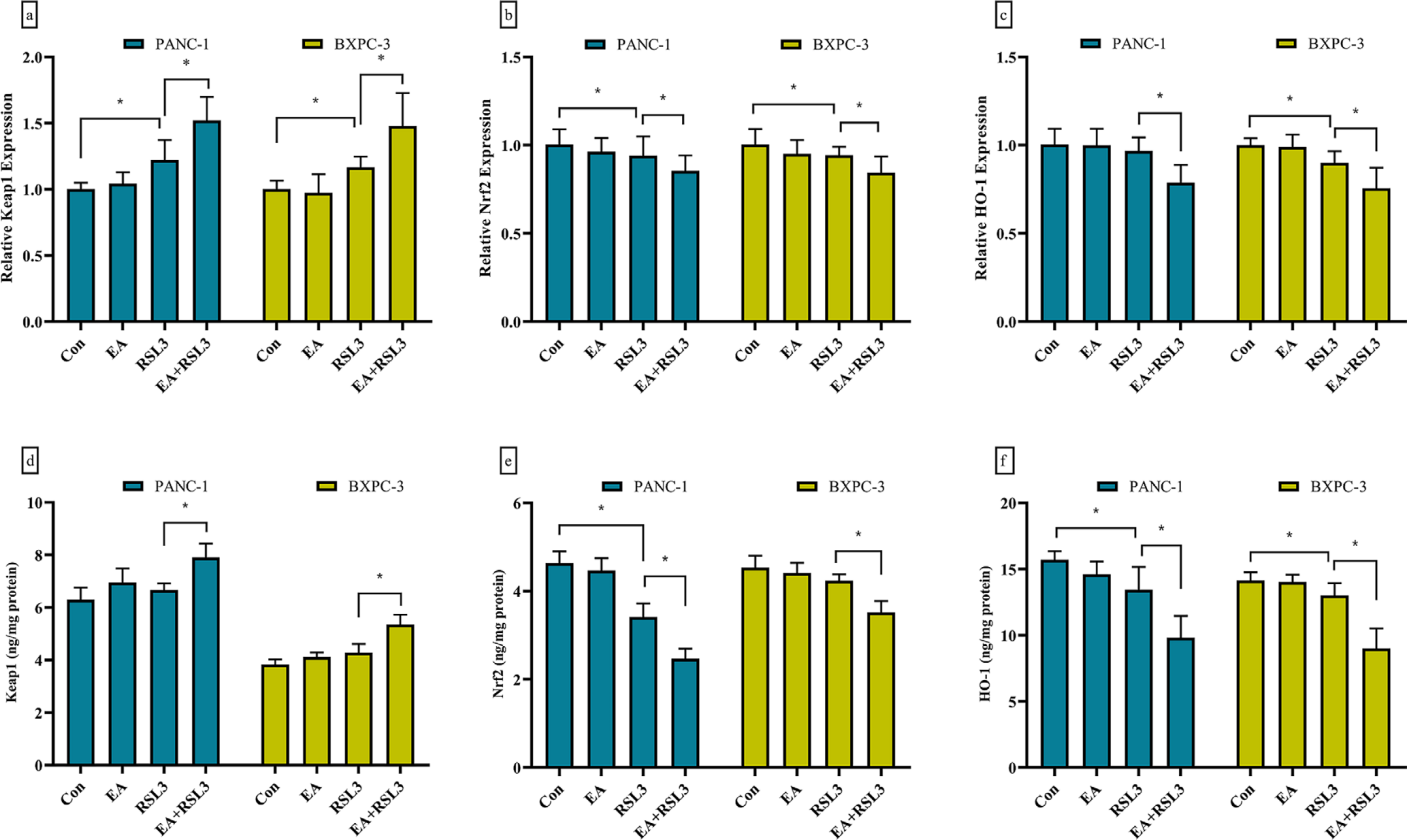
The EA combination with RSL3 regulated the altered expression of Keap1, Nrf2, and HO‐1. (a–c) Gene expression levels of *Keap1*, *Nrf2*, and *HO‐1* were assessed using qRT‐PCR. (d–f) Protein levels of Keap1, Nrf2, and HO‐1 were measured by ELISA. Data are presented as mean ± SD. A *p* < 0.05 was considered significant; *: Indicates a significant difference from controls or the specified group.

To elucidate the molecular mechanisms underlying EA‐mediated enhancement of RSL3‐induced ferroptosis, the gene expression and protein levels of Keap1, Nrf2, and HO‐1 were evaluated in PANC‐1 and BxPC‐3 cells after treatment with EA (50 μM), RSL3 (1 μM), or their combination. In PANC‐1 cells, Keap1 expression was significantly elevated in the EA + RSL3 group (*p* < 0.01), compared to modest increases with EA or RSL3 alone (Figure 3). Similarly, in BxPC‐3 cells, combination therapy robustly elevated Keap1 levels (*p* < 0.001), surpassing monotherapy effects. Concomitant with Keap1 activation, Nrf2 expression was markedly suppressed by EA + RSL3 in both PANC‐1 and BxPC‐3 cells (*p* < 0.001). HO‐1, a downstream target of Nrf2, demonstrated pronounced downregulation in EA + RSL3‐treated cells. In PANC‐1, HO‐1 levels declined to 0.58–0.90‐fold of control (*p* < 0.001), contrasting with minimal changes under EA or RSL3 alone. BxPC‐3 cells exhibited a similar pattern, with combination therapy reducing HO‐1 expression to 0.62–0.89‐fold (*p* < 0.01), while single‐agent treatments showed negligible suppression. Additionally, the analysis of protein levels indicated similar trends in the concentrations of Keap1, Nrf2, and HO‐1 in both studied cell lines (*p* < 0.001).

**FIGURE 4:**
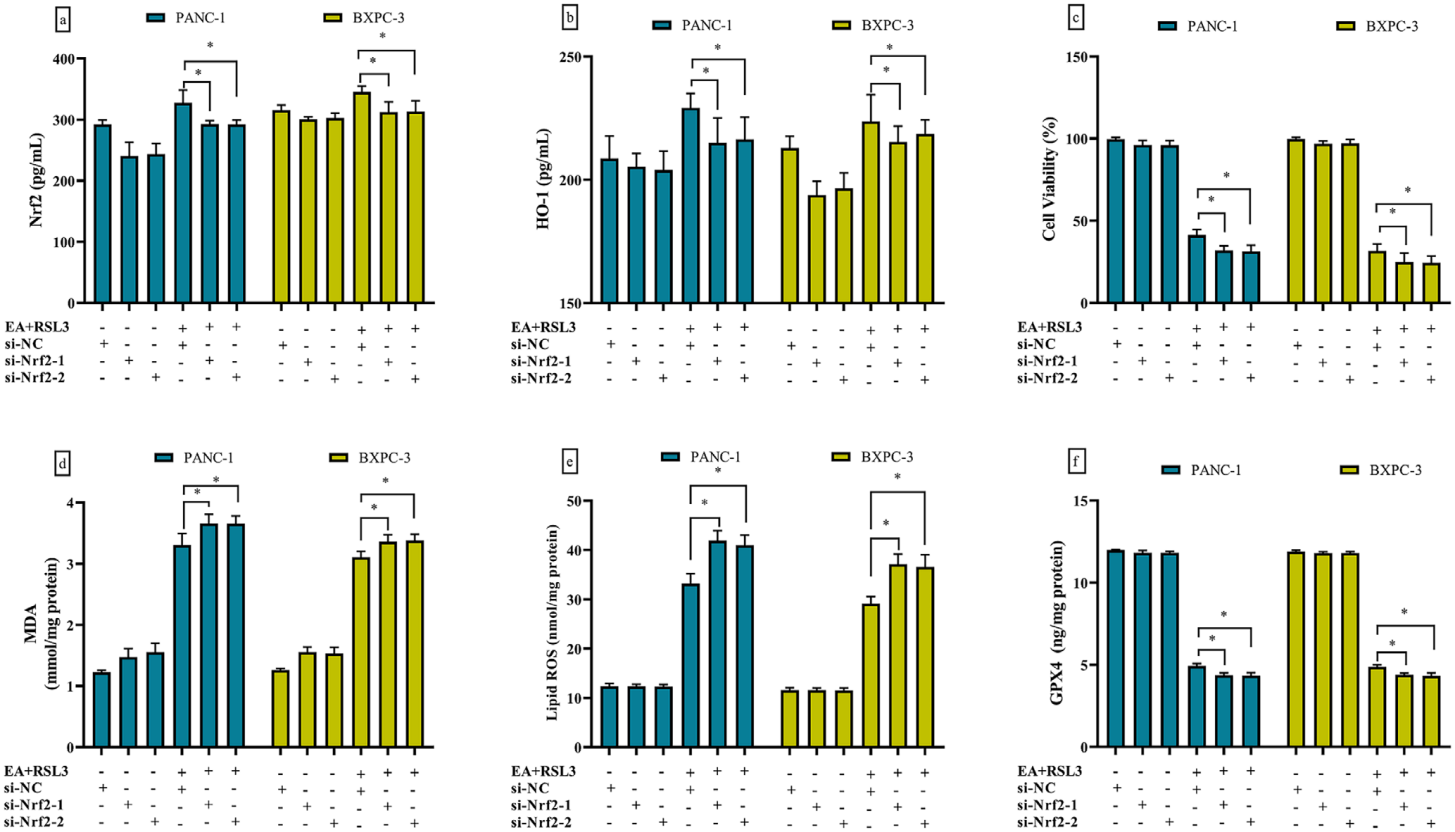
The combination treatment of RSL3 and EA suppresses the Nrf2/HO‐1 axis in PDAC cells. (a) The knockdown of Nrf2 via siRNA resulted in decreased protein levels of Nrf2 following treatment with EA + RSL3 (50 + 1 μM). (b) The knockdown of Nrf2 via siRNA led to reduced protein levels of HO‐1 after treatment with EA + RSL3 (50 μM + 1 μM). (c) siRNA‐mediated Nrf2 knockdown enhanced the sensitivity of PANC‐1 and BxPC‐3 cells to EA + RSL3. (d–f) The levels of MDA, lipid ROS, and GPX4 were quantified in Nrf2‐silenced PANC‐1 and BxPC‐3 cells that were pretreated with or without EA + RSL3. Data are presented as mean ± SD. A *p* < 0.05 was considered significant; *: Indicates a significant difference from controls or the specified group.

To further validate the role of Nrf2, siRNA targeting Nrf2 (si‐Nrf2‐1 and si‐Nrf2‐2) was transfected into PANC‐1 and BxPC‐3 cells. Nrf2 knockdown efficiency was confirmed by reduced Nrf2 protein levels (Figure 4a). Silencing Nrf2 also led to a marked decrease in HO‐1 expression, indicating that HO‐1 is transcriptionally regulated by Nrf2 in PDAC and BxPC‐3 cells (Figure 4b). Nrf2 knockdown significantly enhanced the sensitivity of both PANC‐1 and BxPC‐3 cells to EA + RSL3 treatment (Figure 4c). Subsequently, lipid peroxidation was evaluated in Nrf2‐silenced cells. MDA levels were significantly elevated in si‐Nrf2‐transfected cells treated with EA + RSL3 compared to si‐NC groups (Figure 4d). Concomitantly, lipid ROS levels surged synergistically in Nrf2‐silenced cells treated with EA + RSL3 (Figure 4e). Nrf2 knockdown alone did not alter basal lipid ROS (*p* > 0.05), confirming that Nrf2 ablation specifically sensitizes PDAC cells to ferroptotic lipid peroxidation when GPX4 is inhibited. Furthermore, GPX4 expression, a critical ferroptosis suppressor, was markedly downregulated in Nrf2‐knockdown cells treated with EA + RSL3 (Figure 4f).

### 3.4 EA Stimulates p38 MAPK and Modulates the Nrf2/HO‐1 Pathway

**FIGURE 5:**
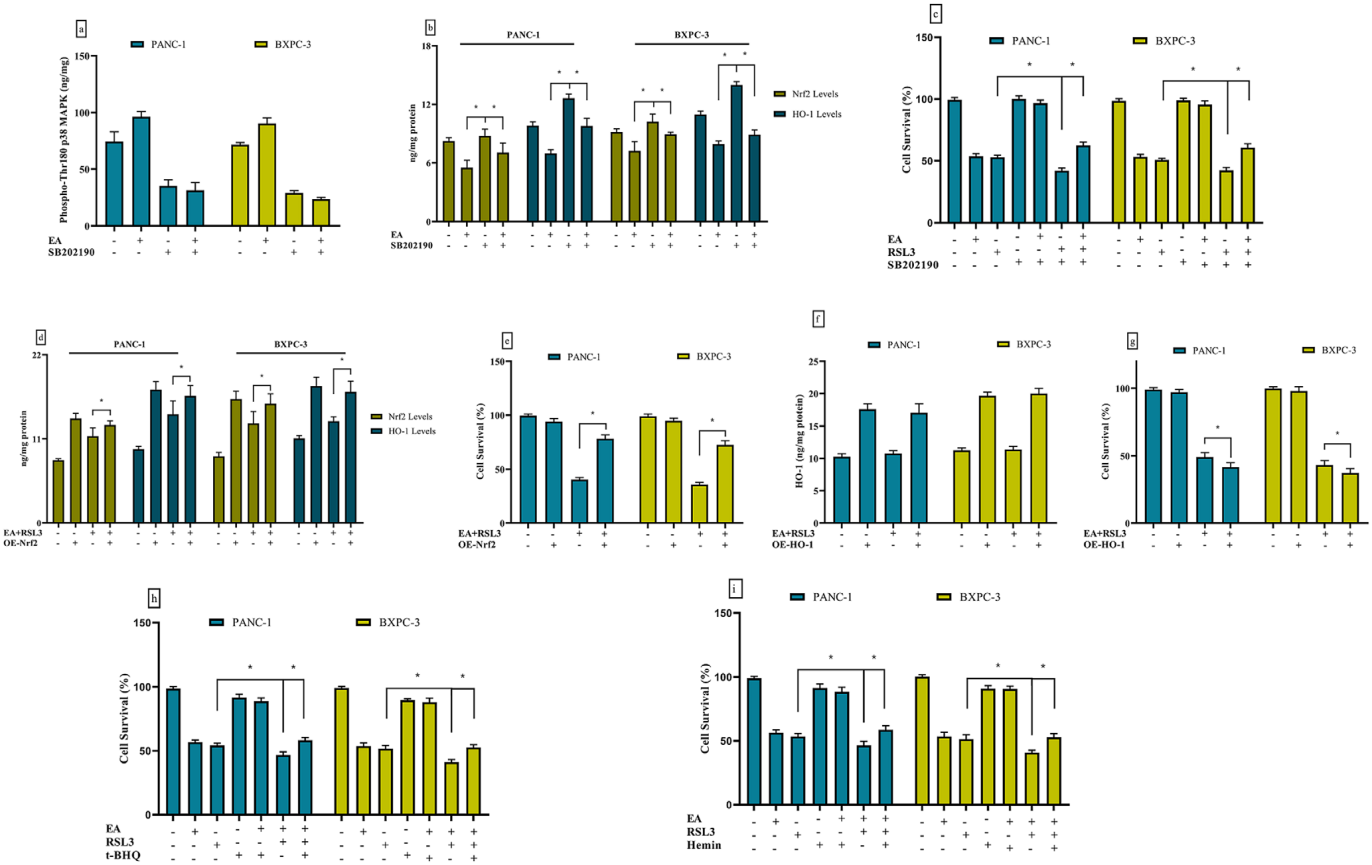
EA stimulates p38 MAPK and modulates the Nrf2/HO‐1 axis. (a, b) The assessment of p‐p38, Nrf2, and HO‐1 protein levels in PANC‐1 and BxPC‐3 cells treated with EA (50 μM), SB202190 (1 μM), or a combination of EA and SB202190. (c) PANC‐1 and BxPC‐3 cells were exposed to EA (50 μM) or RSL3 (1 μM) in the presence or absence of SB202190 (1 μM), and cell viability was evaluated using the CCK‐8 assay. (d) The protein levels of Nrf2 and HO‐1 in PANC‐1 and BxPC‐3 cells, as well as in Nrf2 overexpressing PANC‐1 and BxPC‐3 cells treated with EA + RSL3. (e) PANC‐1 and BxPC‐3 cells with Nrf2 overexpression were treated with or without RSL3 (1 μM) in combination with EA (50 μM), and cell viability was measured using CCK‐8 assays. (f) The protein level of HO‐1 in PANC‐1 and BxPC‐3 cells, or HO‐1 overexpressing PANC‐1 and BxPC‐3 cells treated with EA + RSL3. (g) PANC‐1 and BxPC‐3 cells with HO‐1 overexpression were treated with or without RSL3 (1 μM) in combination with EA (50 μM), and cell viability was evaluated using CCK‐8 assays. (h, i) PANC‐1 and BxPC‐3 cells were treated with EA (50 μM) or RSL3 (1 μM) with or without t‐BHQ (20 μM) or hemin (20 μM), and cell viability was assessed using the CCK‐8 assay. Data are presented as mean ± SD. A *p* < 0.05 was deemed significant; *: Indicates a significant difference from controls or the specified group.

Studies document that p38 MAPK modulates the expression of the antioxidant enzymes, specifically Nrf2 and HO‐1 [40]. To investigate p38 MAPK's role in regulating Nrf2/HO‐1 expression, PANC‐1 and BxPC‐3 cells were treated with EA and the p38‐specific inhibitor SB202190, and their effects on p38 activity were assessed. The levels of phospho‐Thr180 p38 MAPK were significantly increased following EA treatment; however, this enhancement was negated when cells were co‐treated with EA and SB202190 (Figure 5a). Subsequently, this investigation examined whether the inhibition of p38 expression could influence EA‐induced suppression of Nrf2 and HO‐1 expression. ELISA results revealed that EA treatment led to a decrease in the expression of Nrf2 and HO‐1, while SB202190 mitigated the EA‐mediated suppression of these proteins (Figure 5b). Furthermore, the cytotoxicity of PANC‐1 and BxPC‐3 cells induced by RSL3 and EA treatment was partially alleviated by SB202190 (Figure 5c). To further ascertain the contributions of the Nrf2/HO‐1 pathway in the sensitization of cells to RSL3 by EA, PANC‐1 and BxPC‐3 cells were transiently transfected with vectors expressing Nrf2 or HO‐1. As illustrated in Figure 5d,f, the overexpression of Nrf2 reinstated the inhibition of HO‐1 expression and cell proliferation induced by the combination treatment of EA and RSL3 in PANC‐1 and BxPC‐3 cells. Additionally, the overexpression of HO‐1 counteracted the growth‐inhibitory effects of the combined EA and RSL3 treatment in PDAC cells (Figure 5a,g). Moreover, activating Nrf2 (using t‐BHQ) or HO‐1 (using hemin) partially counteracted the inhibitory effects of RSL3 and EA on the growth of PANC‐1 and BxPC‐3 cells (Figure 5h,i). These findings indicate an involvement of p38 MAPK in the regulatory control of Nrf2 and HO‐1 expression, and EA can inhibit the Nrf2/HO‐1 pathway through the activation of p38 MAPK.

### 3.5 EA Promotes Ferroptosis Induced by RSL3 Through the Inhibition of the Nrf2/HO‐1 Pathway in PANC‐1 Cells Within a Xenograft Nude Mouse Model

A xenograft nude mouse model was employed to further examine the role of EA in promoting RSL3‐triggered ferroptosis under in vivo conditions. All mice exhibited good survival rates following cell implantation or treatment with the specified chemicals. After a treatment period, the tumor size progressively increased in both the control and EA treatment groups. In contrast, the administration of RSL3 alone, as well as the combination of both agents, resulted in a reduction in tumor size, with the group receiving both agents demonstrating the most significant decrease in tumor size (*p* < 0.001) and tumor volume (*p* < 0.001). There were no significant changes in body weight or daily food intake observed in either the control or treatment groups (*p* > 0.05). Subsequently, the study examined whether the combined treatment of EA and RSL3 could influence ferroptosis parameters in vivo. Remarkably, total iron, chelatable iron, MDA, lipid ROS, and GPX4 levels were notably altered in the combined treatment group in a way to induce ferroptosis (*p* < 0.001, Table 2).

**TABLE 2:** EA amplifies the inhibitory effects of RSL3 and RSL3‐induced ferroptosis in vivo.

	Con	EA	RSL3	EA + RSL3
Body weight (g)	23.4 ± 0.8	23.6 ± 0.9	21.7 ± 1.3	23.4 ± 0.9
Tumor weight (mg)	3.20 ± 0.11	2.88 ± 0.26*	2.00 ± 0.07*	1.22 ± 0.10*#
Tumor size (mm^3^)	609.6 ± 29.1	578.1 ± 35.3*	404.0 ± 17.1*	312.2 ± 18.3*#
Total iron (μg/g)	45.9 ± 1.6	46.9 ± 1.6	57.6 ± 2.4*	64.9 ± 1.8*#
Labile iron (nmol/mg)	5.8 ± 0.6	6.4 ± 0.5*	7.7 ± 0.2*	8.5 ± 0.2*#
MDA (nmol/mg)	2.9 ± 0.1	3.2 ± 0.2	5.7 ± 0.5*	7.7 ± 0.4*#
Lipid ROS (nmol/mg)	15.1 ± 0.4	16.9 ± 1.3	24.3 ± 2.0*	34.7 ± 2.4*#
GPX4 (ng/mg)	8.33 ± 0.1	7.90 ± 0.2	6.88 ± 0.1*	5.53 ± 0.2*#

*Note:* Data are presented as mean ± SD; *n* = 6 mice per group. *p* < 0.05 was considered significant. *p < 0.05 vs. Con group. # *p* < 0.05 vs. RSL3 group. *# *p* < 0.05 vs. both Con and RSL3 groups.

**FIGURE 6:**
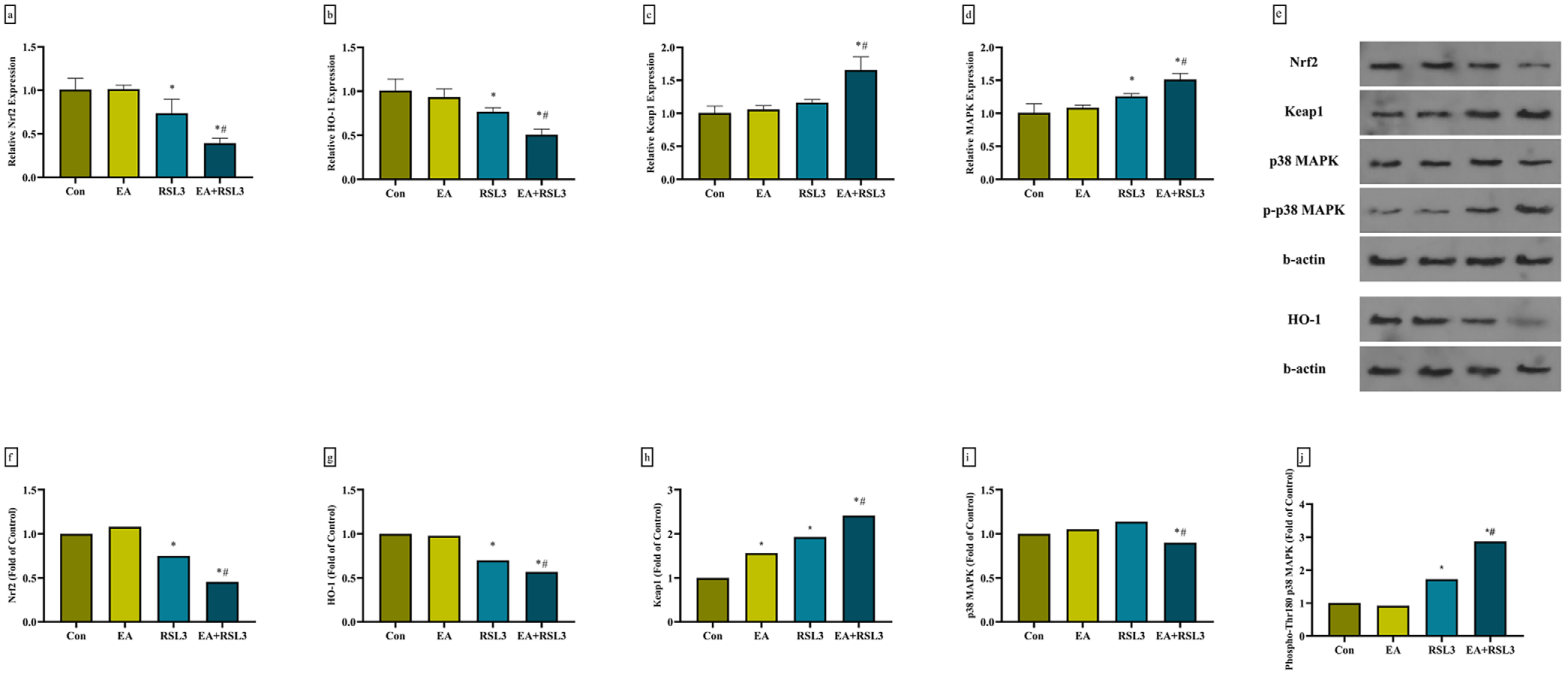
EA promotes RSL3‐induced ferroptosis by modulating the p38/Nrf2/HO‐1 axis and upregulating Keap1 in vivo. (a–d) Gene expression levels of *Nrf2*, *HO‐1*, *Keap1*, and *p38 MAPK*. (e) Western blot analysis of Nrf2, Keap1, p38 MAPK, p‐p38 MAPK, and HO‐1. (f–j) Fold of control levels of Nrf2, HO‐1, Keap1, p38 MAPK, and p‐p38 MAPK. Data are presented as mean ± SD. A *p* < 0.05 was considered significant; *: Indicates a significant difference from controls or the specified group; *#: Indicates a significant difference compared to both control and RSL3 groups; ns, not significant.

Furthermore, the expression levels of Nrf2, HO‐1, Keap1, and p38 MAPK at gene and protein levels were assessed. The qRT‐PCR and western blot results indicated that the relative levels of Nrf2 and HO‐1 were diminished, while Keap1 and phospho‐Thr180 p38 MAPK expression was relatively increased following co‐treatment with RSL3 and EA, consistent with in vitro observations (Figure 6). Notably, the combination therapy revealed the most significant differences when compared to both Con and RSL3 groups (*p* < 0.001).

## 4 Discussion

PDAC remains one of the most lethal malignancies, with reported 5‐year survival rates generally below 10%. This is largely attributable to its aggressive biological behavior, frequent late diagnosis, and intrinsic resistance to conventional therapies. A key contributor to this resistance is the constitutive activation of antioxidant defense systems, particularly the Nrf2/HO‐1 axis, which attenuates oxidative stress and limits the efficacy of standard treatments [41]. While ferroptosis induction, via agents like the GPX4 inhibitor RSL3, holds promise for overcoming apoptosis resistance in PDAC, its efficacy is frequently limited by adaptive Nrf2‐mediated antioxidant upregulation [23]. Concurrently, despite evidence that natural compounds like EA exert multi‐targeted anticancer effects, their role in modulating ferroptosis sensitivity remains underexplored. To address these gaps, this study was designed to investigate whether EA potentiates RSL3‐induced ferroptosis in PDAC models, elucidate the mechanistic involvement of the p38 MAPK/Nrf2/HO‐1 signaling axis in this synergy, and evaluate the translational potential of EA‐RSL3 co‐treatment in vivo.

The present findings demonstrate that co‐treatment with EA and RSL3 synergistically triggers ferroptotic cell death in PDAC models. In vitro, the combination regimen markedly amplified hallmark ferroptotic events, including intracellular iron accumulation, lipid peroxidation (elevated MDA and lipid ROS), and suppression of the key ferroptosis defense enzyme GPX4. Critically, ferroptosis‐specific inhibition (Fer‐1), but not blockers of apoptosis, necroptosis, or autophagy, rescued cell viability, confirming ferroptosis as the dominant death mechanism. These observations were recapitulated in vivo, where EA + RSL3 co‐administration significantly suppressed xenograft tumor growth without systemic toxicity, concomitant with increased tumor tissue iron, MDA, and lipid ROS, alongside downregulation of GPX4. This aligns with prior reports that KRAS‐mutant cancers exhibit heightened susceptibility to ferroptosis inducers due to altered iron metabolism and redox homeostasis. For instance, Yang et al. reported that cetuximab potentiated RSL3‐induced ferroptosis in KRAS‐mutant CRC by suppressing Nrf2/HO‐1. However, this work extends this paradigm in several key aspects. First, this study identified a natural compound, not a monoclonal antibody, as the Nrf2/HO‐1 suppressor, offering a complementary therapeutic strategy. Second, this investigation established this synergy in PDAC, a malignancy with even greater stromal complexity and resistance than CRC. Finally, it was elucidated that p38 MAPK activation is the possible upstream mechanism by which EA disrupts the Nrf2/HO‐1 axis, providing a novel mechanistic layer absent in prior studies. Similar ferroptotic responses observed in both cell culture and xenograft models support the reproducibility of the EA + RSL3 effect. Notably, EA exhibits a dual redox‐modulating role; while it acts as a pro‐oxidant in cancer cells to promote ferroptosis, it functions as an antioxidant in normal tissues [42, 43]. This selectivity may arise from differential basal redox states, where malignant cells, already under heightened oxidative stress, are pushed beyond a survivable threshold by EA‐induced Nrf2/HO‐1 suppression. In contrast, normal cells leverage EA's antioxidant properties to bolster endogenous defenses, explaining its favorable safety profile in preclinical models [44, 45]. These observations suggest that EA may enhance ferroptosis in tumor cells while exerting limited oxidative toxicity in normal tissues.

Our data indicate that the p38 MAPK/Nrf2/HO‐1 axis contributes to the ferroptotic interaction between EA and RSL3. In alignment with earlier studies that connect p38 signaling to the modulation of Nrf2‐dependent antioxidant responses, treatment with EA resulted in an increase in p38 phosphorylation while concurrently reducing the expression of Nrf2 and HO‐1. The pharmacological inhibition of p38 or the restoration of Nrf2/HO‐1 signaling diminished the ferroptotic response, thereby reinforcing the significance of p38‐mediated suppression of the Nrf2/HO‐1 pathway in enhancing the sensitivity of PDAC cells to ferroptosis [46, 47]. Moreover, in addition to the p38‐dependent regulation, the combination of EA and RSL3 also led to an elevation in Keap1 expression, which may further inhibit Nrf2 signaling and strengthen ferroptotic susceptibility. Comparable regulatory interactions between Keap1–Nrf2 signaling and sensitivity to ferroptosis have been documented in various cancer models [48, 49]. Additionally, KRAS mutation status, present in both studied cell lines, may intrinsically rewire iron metabolism through MAPK pathway hyperactivation, as suggested by elevated basal iron in cells versus H6C7 controls.

From a therapeutic perspective, these findings suggest that EA may act as a ferroptosis‐sensitizing agent in PDAC. Beyond its favorable safety profile as a dietary polyphenol, a key therapeutic advantage of EA lies in its polypharmacological nature. Unlike monoclonal antibodies or synthetic kinase inhibitors that typically target a single protein, EA concurrently modulates multiple signaling nodes, activating stress‐responsive p38 MAPK and upregulating the Nrf2 repressor Keap1 to cooperatively suppress the Nrf2/HO‐1 axis. This multi‐target approach provides a more potent and sustained disruption of PDAC's antioxidant defense network, potentially reducing the likelihood of compensatory resistance mechanisms often encountered with highly specific targeted therapies. While its established safety profile offers translational advantages, it is this synergistic dual‐pathway inhibition that makes the EA + RSL3 combination particularly compelling for overcoming the redundant signaling inherent to PDAC. Although the present study did not evaluate pharmacokinetic parameters, these characteristics should be carefully considered when translating EA‐based combinations into clinical settings. Previous studies indicate that EA exhibits relatively limited systemic bioavailability due to rapid metabolism and its conversion into bioactive urolithin derivatives by intestinal microbiota. These metabolites may contribute substantially to the biological activity observed in vivo and could influence therapeutic responses in clinical settings. Furthermore, components of the p38–Keap1–Nrf2–HO‐1 signaling axis may represent potential predictive biomarkers for ferroptosis‐based therapies. Because Nrf2 signaling and HO‐1 expression are closely linked to cellular redox homeostasis and ferroptosis resistance, tumor expression levels of these molecules may help identify PDAC patients who are more likely to benefit from strategies combining EA with ferroptosis inducers such as RSL3. Future translational studies integrating pharmacokinetic profiling and biomarker stratification will therefore be important for optimizing the clinical development of this combinatorial approach [50, 51, 52].

Despite the promising antitumor effects observed in this study, several limitations should be considered. Our in vivo experiments were conducted using a subcutaneous xenograft model, which, although widely used for evaluating tumor growth and therapeutic responses, does not fully recapitulate the complex tumor microenvironment, stromal interactions, and anatomical context of pancreatic ductal adenocarcinoma. Future studies employing orthotopic PDAC models or patient‐derived organoid systems will be valuable to further validate the therapeutic potential and mechanistic relevance of the EA‐RSL3 combination in a setting that more closely reflects human disease. Future studies should evaluate EA + RSL3 with standard chemotherapies (gemcitabine/nab‐paclitaxel) to assess broader synergy and interrogate stromal crosstalk using co‐culture models with cancer‐associated fibroblasts. Moreover, research on profile biomarkers (e.g., circulating lipid peroxides) is encouraged to identify patient subgroups most likely to benefit. Despite these constraints, these findings provide mechanistic insight into how natural compounds such as EA may help overcome redox‐associated resistance in PDAC.

## 5 Conclusion

This study demonstrates that ellagic acid synergistically enhances RSL3‐induced ferroptosis in PDAC models by dual modulation of the p38 MAPK/Nrf2/HO‐1 signaling axis and Keap1 upregulation. In vitro, EA co‐treatment amplified hallmark ferroptotic events such as iron accumulation, lipid peroxidation, and GPX4 suppression, while in vivo, it significantly inhibited tumor growth. Mechanistically, EA activated p38 MAPK, leading to Nrf2/HO‐1 downregulation, and concurrently elevated Keap1, promoting Nrf2 degradation. These findings position EA + RSL3 as a promising combinatorial strategy against therapy‐resistant PDAC. Clinical translation should evaluate EA's pharmacokinetics and long‐term safety to harness its potential in overcoming redox adaptation in pancreatic cancer.

## Author Contributions

Jianhua Bai, Yihe Dai, Chern Ein Oon, Amirabas Bostani, Yun Jin, and Zhenhao Fei were responsible for the conception and design of the study. Pingping Hu, Jia Luo, Jiang Han, and Liman Yang executed the experiments and conducted the data analysis. Jianhua Bai and Yihe Dai drafted the initial manuscript. All authors reviewed and approved the final manuscript version.

## Funding

Famous Doctor Projects of Yunnan Province (XDYC‐MY‐2022‐0032); Liu Liang Expert Workstation of Yunnan Province (202305AF150148); Yunnan health training project of high level talents (L‐2024029).

## Disclosure

Declaration of Generative AI in Scientific Writing: During the preparation of this work, the authors used the DeepSeek tool in order to paraphrase the text. After using this tool/service, the authors reviewed and edited the content as needed and take full responsibility for the content of the published article.

## Ethics Statement

The current study was designed and conducted in accordance with international guidelines pertaining to animal welfare and the National Institute of Health (NIH). The use of animals and the procedures involved in this study were evaluated and sanctioned by the local ethics committee (Ethics Committee of Beijing Long'an Laboratory Animal Breeding Center, GENINK‐20250708) and were carried out in compliance with ARRIVE guidelines.

## Conflicts of Interest

The authors declare no conflicts of interest.

## Supporting information


**Data S1:** fsb272166‐sup‐0001‐DataS1.docx.

## Data Availability

The datasets utilized and/or examined during this study are submitted along with the manuscript and also can be obtained from the corresponding author.
