# Supplementary material for: Ellagic Acid Enhances RSL3‐Induced Ferroptosis by Inhibiting the Nrf2/HO‐1 Signaling Pathway in Pancreatic Ductal Adenocarcinoma
Source: FASEB J. 2026 Sep 18;40(18):e72166. doi: 10.1096/fj.202602231R (PMC13587256; doi:10.1096/fj.202602231R)
Supplement: Supplementary file 1 [file FSB2-40-e72166-s001.docx]

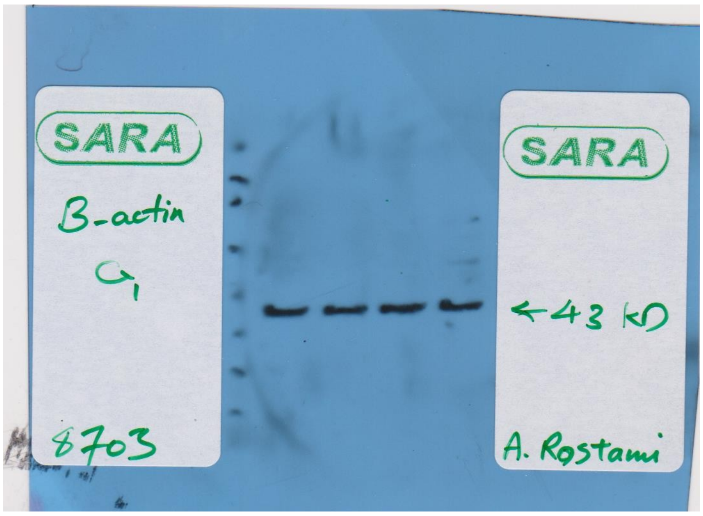
SARA
B-actin
G1
8703
SARA
←43 kD
A. Rostami

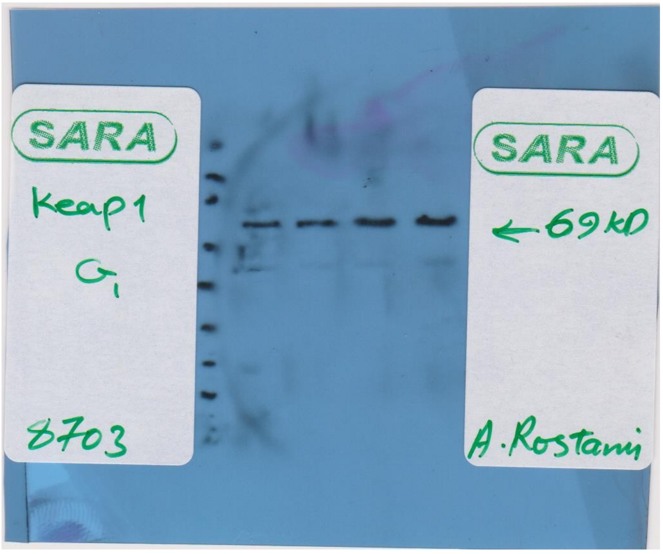
SARA
Keap1
G1
8703
SARA
←69 kD
A. Rostami

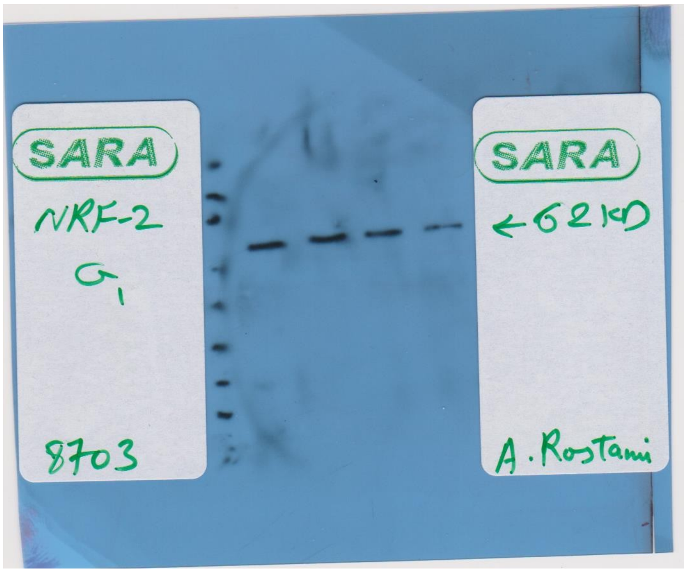
SARA
NRF-2
G1
8703
SARA
←62 kD
A. Rostami

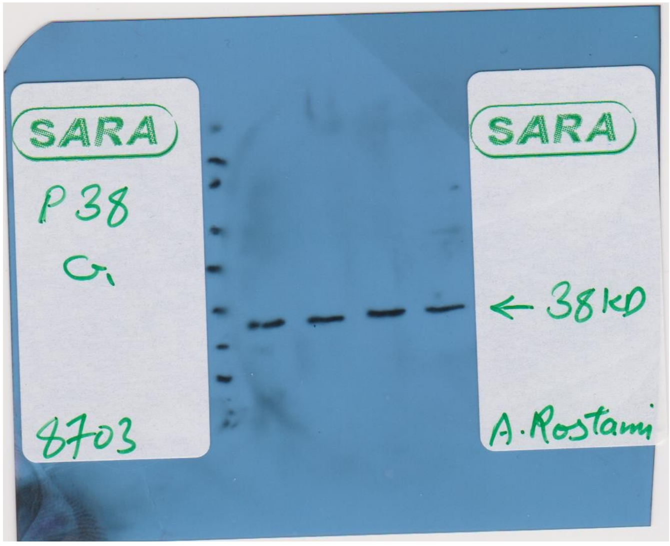
SARA
P38
G1
8703
SARA
← 38KD
A.Rostami

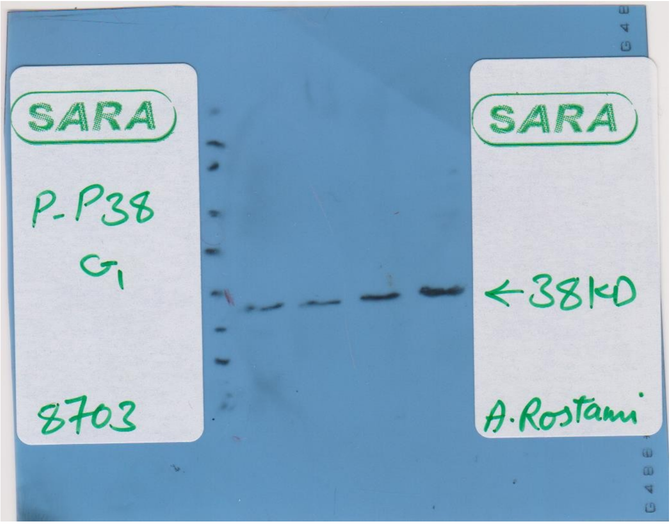
SARA
P-P38
G1
8703
SARA
← 38KD
A.Rostami

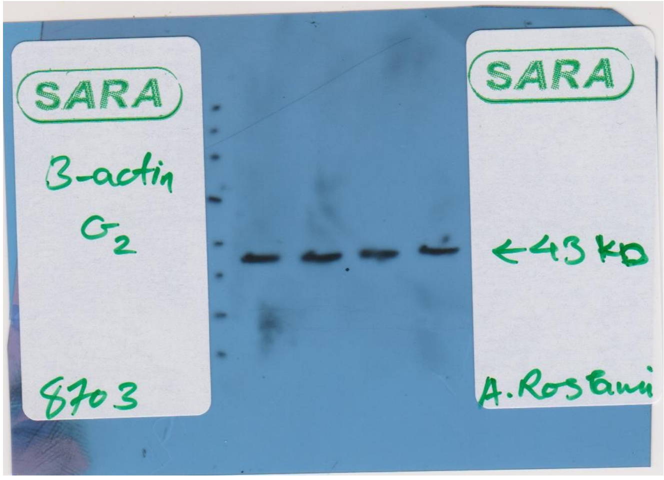
SARA
β-actin
G2
8703
SARA
← 43 KD
A.Rostami

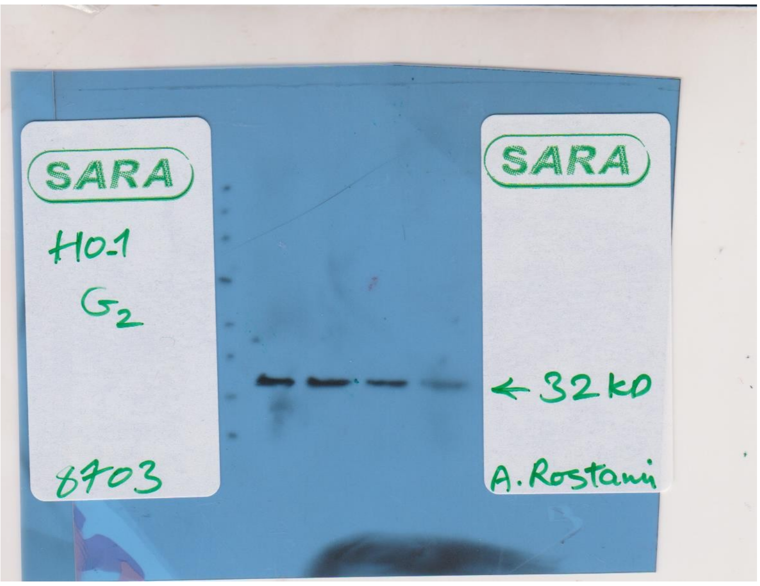
SARA
HO-1
G2
8703
SARA
← 32 kD
A. Rostami
